# NBD-Based Environment-Sensitive Fluorescent Probes for the Human Ether-a-Go-Go–Related Gene Potassium Channel

**DOI:** 10.3389/fmolb.2021.666605

**Published:** 2021-05-14

**Authors:** Qi Li, Lijuan Chai, Gaopan Dong, Xiaomeng Zhang, Lupei Du

**Affiliations:** ^1^ Department of Medicinal Chemistry, Key Laboratory of Chemical Biology (MOE), School of Pharmacy, Shandong University, Jinan, China; ^2^ Department of Internal Medicine, Hospital of Shandong University, Jinan, China

**Keywords:** hERG potassium channel, fluorescent labeling technology, small-molecule fluorescent probe, fluorescent imaging, cell imaging

## Abstract

Three environment-sensitive probes were developed for the hERG channel based on the nitrobenzoxadiazole fluorophore herein. After careful evaluation, probes **M1** and **M3** were found to have a high affinity for imaging the hERG channel in the cell-based experiment. Compared with other fluorescent labeling technologies (such as fluorescent proteins), these probes afford a convenient and economical method to determine hERG channel *in vitro* and *in cellulo*. Therefore, these probes are expected to be applicable for usage in physiological and pathological studies of hERG channels and have the potential to establish a screening system for hERG channels.

## Introduction

The human ether-a-go-go–related gene (hERG) codes to form a rapidly delayed rectifying potassium ion channel. The channel consists of four identical alpha subunits forming a tetrameric structure and a pore-forming region in the middle. Each subunit consists of six helical transmembrane domains (denoted as S1–S6) (Doyle et al., 1998). In cardiomyocytes, loss of hERG function is one of the causes of acquired long QT interval syndrome, and acquired functional mutation may also cause short QT interval syndrome (Moss et al., 2002). At present, many drugs that are on the market or in the clinical stage have prolonged the QT interval due to their inhibitory effect on the hERG channel and are withdrawn from the market. Prolonged QT interval displays delayed ventricular repolarization, which can cause *torsades de pointes* (TdP), thus leading to cardiotoxicity. Therefore, the hERG potassium channel is currently the main anti-target. At the FDA's request, all drugs need to be tested for their affinity for hERG channels to assess their cardiotoxicity (Raschi et al., 2008; Liu et al., 2016).

The hERG potassium channel distributes in various cancer cells, neurons, smooth muscle cells of organs, and chromaffin cells, performing different roles in different tissues (Raschi et al., 2008). Over the past 2 decades, there is increasing evidence that the hERG potassium channel abnormally expresses in tumor cell lines and primary human cancers, such as gliomas, neural crest–derived tumors. Cell and molecular studies indicate that the hERG channel regulates different aspects of tumor progression, such as cell proliferation and survival, secretion, invasion, and metastasis of pro-angiogenic factors (Jehle et al., 2011). Therefore, the hERG potassium channel may be the target for particular cancer treatment. To better analyze and determine the hERG potassium channel at the molecular and cellular level, developing a simple, cost-effective, and safe fluorescent method for labeling the hERG potassium channel is urgently required (Luo et al., 2020). Effective development of such fluorescent labeling may explain the hERG channel's role in those cancers and anatomical locations, thus establishing the hERG channel as a brand new target and/or biomarker for antitumor agents.

The fluorescent labeling technologies for the hERG potassium channel mainly rely on small-molecule fluorescent probes and fluorescent protein–based labeling technologies (Claassen et al., 2008; Huang et al., 2011; Mizukami and Kikuchi, 2016; Huang et al., 2020). With the unique characteristics such as high sensitivity, low detection limit, simple preparation, and low cost, the application of small-molecule fluorescent probe is available for detecting and imaging enzymes, receptors, ion channels, DNA, RNA, and biologically active small molecules (H_2_S, H_2_O_2_, etc.) directly, or for even tracking dynamic processes in a real-time manner at the animal level (Chan et al., 2012; Mizukami et al., 2013).

After the fluorescent probe binds to the target, the fluorophore converts the interaction between the recognition and target groups into a fluorescent signal. In this process, the excess unbound fluorescent probe has a specific effect on target binding and fluorescence detection (Mizukami et al., 2013). An inevitable turn-on switch is usually incorporated into the design of the probe. It is known that the principal mode of action between ligands and the hERG potassium channel is through hydrophobic interaction with Tyr 652 and/or Phe 656 residues. Therefore, we suspect that an environment-sensitive fluorophore, such as 6-dimethylaminonaphthalene (DAN), 1,8-anilinonaphthalenesulfonic acid (ANS), nitrobenzoxadiazole (NBD), and sulfonyl benzoxadiazole (SBD), can be integrated into the probe design (Environment-Sensitive Fluorescent Probe for the Human Ether-a-go-go-Related Gene Potassium Channel, Environment-Sensitive Fluorescent Turn-On Probes Targeting Hydrophobic Ligand-Binding Domains for Selective Protein Detection, Kilpin et al., 2012; Zhuang et al., 2013). Considering that the probe's bulky size may affect the affinity with the target, we chose a relatively small NBD fluorophore to participate in the hydrophobic interaction with the hERG channel as a part of the recognition group (Ma et al., 2016; Perez et al., 2019).

## Results and Discussion

### Design and Synthesis of Fluorescent Probes

Astemizole and E-4031 are both very effective drugs with inhibitory effects on the hERG channel (Zhou et al., 1999; Tian et al., 2017; Dickson et al., 2020). Studies on the mechanism of action between the hERG potassium channel and inhibitors have revealed that the high-affinity binding of hERG channels requires the inhibitor’s aromatic group and basic nitrogen. Based on the above, the major interacting parts of astemizole and E-4031 were selected as the recognition group.

**SCHEME 1:**
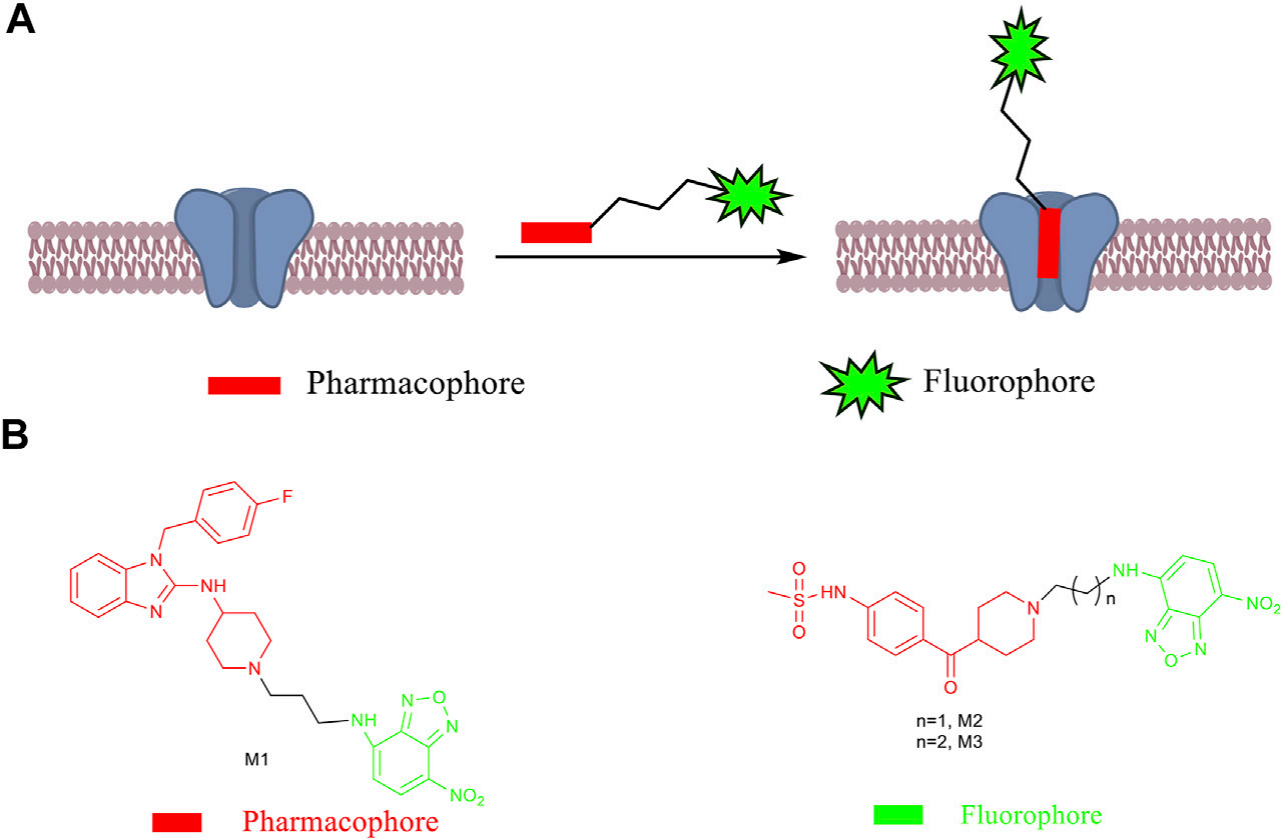
**(A)** Design principle of fluorescent probes. **(B)** Chemical structures of small-molecule fluorescent probes **M1**–**M3**.

**FIGURE 1:**
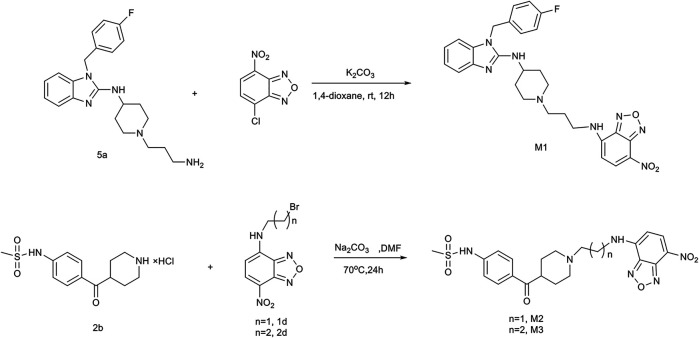
Synthetic routes of fluorescent probes **M1**, **M2**, and **M3**.

As depicted in Scheme 1 and Figure 1, probes **M1**, **M2**, and **M3** were well designed and synthesized through several convenient steps. In brief, the fluorophore NBD-Cl and astemizole-based intermediate **5a** produced probe **M1** in the presence of K_2_CO_3_ in 1,4-dioxane. NBD-Cl derivatives **1d** and **2d** reacted with E-4031–based intermediate **2b** to provide probes **M2** and **M3** in DMF in the presence of NaCO_3_. Detailed information on synthetic intermediates can be found in the supporting information.

### Spectroscopic Properties of Fluorescent Probes

**FIGURE 2:**
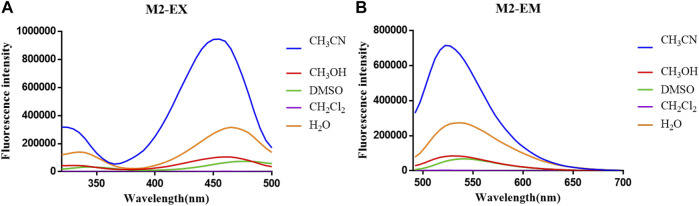
**(A)** Fluorescent excitation and **(B)** emission spectra of probe **M1** in different solvents.

**FIGURE 3:**
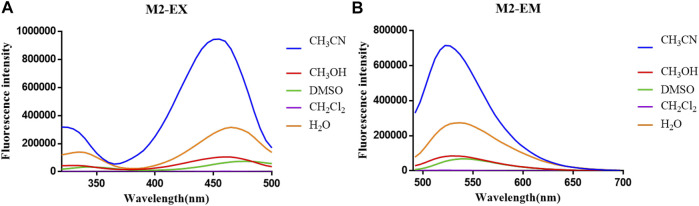
**(A)** Fluorescent excitation and **(B)** emission spectra of probe **M2** in different solvents.

**FIGURE 4:**
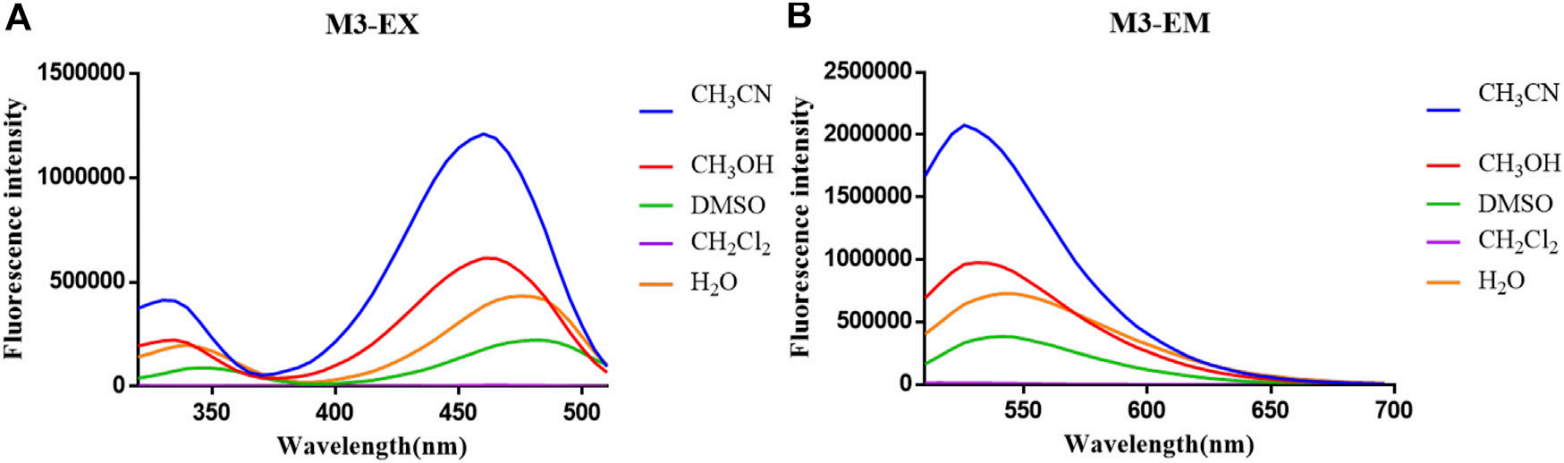
**(A)** Fluorescent excitation and **(B)** emission spectra of probe **M3** in different solvents.

The optical properties of probes **M1**, **M2**, and **M3** were measured in a 10 μM PBS solution (pH = 7.4). The experimental results display that these probes have rational optical properties (Table 1). Moreover, the varied fluorescence intensity in different solvents indicates the environment-sensitive effect caused by the NBD-Cl fluorophore (Figures 2–4).

**TABLE 1:** Optical properties of probes **M1**, **M2**, and **M3**.

Probes	*λ*_max_ (nm)	*ε* (L/mol.cm)	*λ*_ex_ (nm)	*λ*_em_ (nm)	*Ф* (%)
**M1**	475	6.06 × 10^4^	475	545	9.40
**M2**	465	3.07 × 10^4^	470	530	7.31
**M3**	470	5.10 × 10^4^	475	545	10.1

### Cytotoxicity Assay of Fluorescent Probes

The cytotoxicity of probes **M1**–**M3** for hERG-transfected HEK293 cells was evaluated by an SRB assay. The experimental results showed that these probes have low cytotoxicity, have little effect on the viability of living cells, and can be used to detect and image hERG potassium channels in living cells (Table 2).

**TABLE 2:** Cytotoxicity of fluorescent probes for hERG-transfected HEK293.

Probes	IC_50_ (μM)
	hERG-HEK 293
Astemizole	17.4 ± 1.06
**M1**	10.6 ± 2.74
**M2**	>100
**M3**	>100

### Binding Affinity of Fluorescent Probes

Cell membranes extracted from hERG-transfected HEK293 cells were evaluated for probes' affinity to the hERG channel by a radioligand binding assay. The binding results demonstrated that probe **M1** exhibited a high affinity for hERG potassium channels. The calculated IC_50_ and K_i_ values were 6.75 and 3.79 nM, respectively, slightly lower than those for astemizole (4.76 and 2.67 nM, respectively). The affinity of probe **M3** for the hERG channel is similar to that of **M1**, with IC_50_ of 10.8 nM and K_i_ of 6.08 nM. Although having lower activity than probe **M1** and astemizole, probe **M2** still displays a potent affinity for the hERG channel (Table 3).

**TABLE 3:** Affinity of probes for hERG-transfected HEK293.

Molecule	IC_50_ ^a^ (nM)	K_i_ ^b^ (nM)
Astemizole	4.76	2.67
**M1**	6.75	3.79
**M2**	67.4	37.8
**M3**	10.8	6.08

a See the Supporting Information.

b The inhibition constant (Ki) was calculated from each IC_50_ value using the Cheng–Prusoff equation.

### Fluorescent Imaging Studies

Considering their sufficient affinity for hERG potassium channel, good fluorescent properties, and suitable cytotoxicity, probes **M1**–**M3** were applied for labeling the hERG channel in living cells. Initially, the autofluorescence of hERG-HEK293 cells was well evaluated. The experimental results indicated that the cell autofluorescence is too weak to be measured with or without astemizole (Supplementary Figure S2). Therefore, this weak cell autofluorescence would not interfere with the labeling of the hERG channel by probes **M1**–**M3**.

**FIGURE 5:**
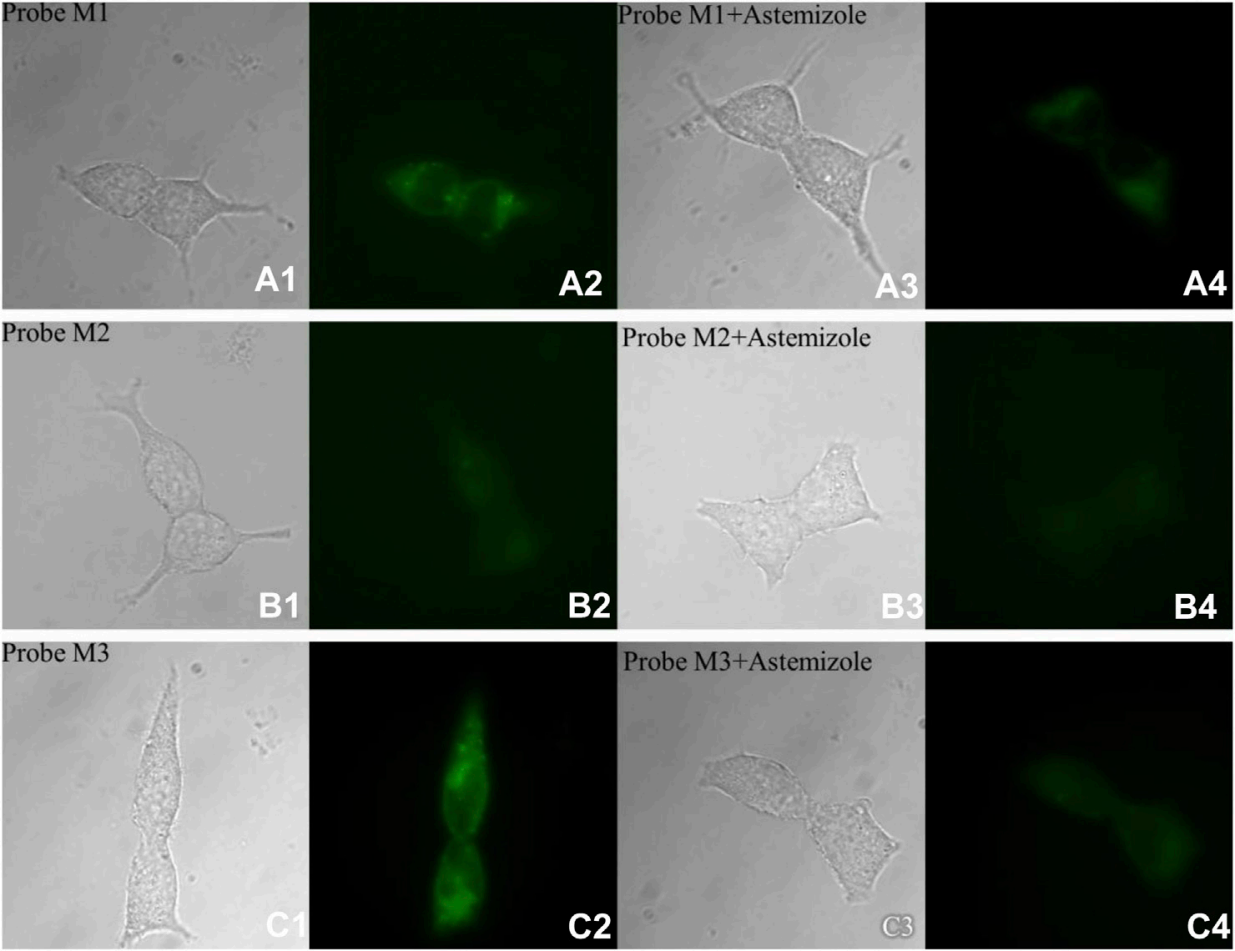
Fluorescence microscopy imaging of hERG-transfected HEK293 cells incubated with 1.0 μM probe **M1** (**A1**: bright field; **A2**: GFP channel), 5.0 μM probe **M2** (**B1**: bright field; **B2**: GFP channel), and 5.0 μM probe **M3** (**C1**: bright field; **C2**: GFP channel). The imaging of inhibition of the hERG channels was accomplished by incubating astemizole (10, 50, and 50 μM) with probes **M1** (1.0 μM; **A3**: bright field; **A4**: GFP channel), **M2** (5.0 μM; **B3**: bright field; **B4**: GFP channel), and **M3** (5.0 μM; **C3**: bright field; **C4**: GFP channel). All cells were incubated with each probe at 37°C for 10 min and washed immediately. The background was adjusted by ImageJ software. Imaging was performed using a Zeiss Axio Observer A1 microscope with a ×63 objective lens. Scale bar = 20 μm.

In cell imaging, probe **M1** can achieve high brightness at low concentrations (0.1 μM), consistent with its affinity from the radioligand binding experiment. The reason why probes **M2** and **M3** require high concentrations (5 μM) is that the pharmacophore of probes **M2** and **M3** is E-4031, which has a slightly lower affinity for hERG channels than astemizole. Moreover, a potent hERG channel inhibitor (astemizole) was selected to incubate cells with each probe. After incubation with astemizole, the fluorescence intensity of all probes decreased significantly (Figure 5, Table 4).

**TABLE 4:** Fluorescence intensity of cell imaging.

Molecule	Mean^a^ (probe)	Mean^a^ (probe + astemizole)
**M1**	51.076	34.666
**M2**	34.988	28.353
**M3**	67.015	26.213

a Mean: Mean gray value; Mean = IntDen/Area

## Conclusion

In conclusion, we designed, synthesized, and evaluated three small-molecule fluorescent probes herein. Introduction of the environment-sensitive fluorophore NBD revealed that these probes do not require a time-consuming washing procedure during microscopic imaging. Compared with other reported fluorescent labeling technologies, such as fluorescent proteins, these probes provide a convenient and affordable method for detecting the hERG channel. It should be noted that among all probes, molecules **M1** and **M3** have higher affinity and more vigorous fluorescence intensity than our previously published environment-sensitive probes (Environment-Sensitive Fluorescent Probe for the Human Ether-a-go-go-Related Gene Potassium Channel, Wang et al., 2016). However, more challenge remains to be undertaken, especially in developing near-infrared probes for hERG channel for *in vivo* research.

## Data Availability

The datasets presented in this study can be found in online repositories. The names of the repository/repositories and accession number(s) can be found in the article/Supplementary Material.
