# Supplementary material for: NBD-Based Environment-Sensitive Fluorescent Probes for the Human Ether-a-Go-Go–Related Gene Potassium Channel
Source: Front Mol Biosci. 2021 May 14;8:666605. doi: 10.3389/fmolb.2021.666605 (PMC8160426; doi:10.3389/fmolb.2021.666605)
Supplement: Supplementary file 1 [file DataSheet1.docx]

Supplementary Material

**NBD-based environment-sensitive fluorescent probes for the Human Ether-a-go-go-Related Gene Potassium Channel**

Qi Li,^1,#^ Lijuan Chai,^2,#^ Gaopan Dong,^1^ Xiaomeng Zhang,^1^ and Lupei Du^1^ (✉)

^1^Department of Medicinal Chemistry, Key Laboratory of Chemical Biology (MOE), School of Pharmacy, Shandong University, Jinan, Shandong 250012, China
^2^Department of Internal Medicine, Hospital of Shandong University, Jinan, Shandong 250012, China

*** Corresponding author:** Lupei Du: [dulupei@sdu.edu.cn](mailto:dulupei@sdu.edu.cn)

# These authors contributed equally.

[1. Synthesis 2](#_Toc18926)

[2. Fluorescent Properties of Probes Assay 9](#_Toc21102)

[3. Cytotoxicity 10](#_Toc31734)

[4. Microscopic imaging of hERG-HEK293 cells(Liu et al. 2015) 11](#_Toc3324)

[5. 1H-NMR, 1^3^C-NMR, ESI-HRMS 11](#_Toc27902)

[6. References 15](#_Toc8167)

1. **Synthesis**

All reagents and solvents available from commercial sources were used as received unless otherwise noted. Water used for the fluorescence studies was doubly distilled and further purified with a Mill-Q filtration system. ^1^H NMR and ^13^C NMR were recorded on a Bruker 400 MHz NMR spectrometer. Mass spectra were performed by the analytical and the mass spectrometry facilities at Shandong University and Shandong Provincial Academy of Sciences. Absorption spectra and fluorescence spectra were obtained with a PerkinElmer EnSight microplate reader. Fluorescence imaging was performed using a Zeiss Axio Observer A1 fluorescence microscopy.

**Scheme S1.** Synthesis of astemizole-based pharmacophore

**Synthesis of 2-chloro-1-(4-fluorobenzyl)-1H-benzo[d]imidazole (1a)**

A mixture of 2-chlorobenzimidazole (1.0 g, 6.55 mmol) and potassium hydroxide (0.52 g, 9.27 mmol) was mixed in 10 mL of acetonitrile, and the mixture was refluxed at 80 ° C for 30 minutes until the solution was clear. After cooling to room temperature, p-fluoroammonium bromide (1.86 g, 9.82 mmol) was added, and after stirring well, refluxed at 80 ° C for 10 h. After the reaction, suction filtration was used to collect the filtrate. The filtrate was dried under vacuum. The crude product was purified by column chromatography to obtain a white solid powder 1.32 g in yield 77.6%, mp: 67 -70 ℃

^1^H NMR (400 MHz, CDCl_3_) *δ* 7.76 – 7.69 (*m*, 1H), 7.30 – 7.28 (*m*, 1H), 7.27 (*d, J* = 3.1 Hz, 1H), 7.24 *(t, J* = 2.8 Hz, 1H), 7.17 (*dd, J* = 8.5, 5.2 Hz, 2H), 7.02 (*t, J* = 8.6 Hz, 2H), 5.37 (*d, J* = 5.8 Hz, 2H).

ESI-MS：([M+H]^+^) calcd for C_14_H_10_ClFN_2_：261.1，found：261.2.

**Synthesis of Ethyl-4-((1-(4-fluorobenzyl)-1H-benzo[d]imidazol-2-yl)amino)piperidine-1 carboxylate (2a)**

In a 100 mL Schlenk tube, a mixture of **1a** (1.32 g, 5.06 mmol) and ethyl 4-amino-1-piperidinecarboxylate (1.31 g, 7.60 mmol) was stirred at 170 ° C for 8 h. After the reaction was completed, the reaction solution was brown. After the reaction, cooled to room temperature, added 20 mL of dichloromethane and 0.5 mL of anhydrous methanol, and sonicated until the reaction solution was completely dissolved. The crude product was purified by column chromatography to obtain **2a** as a light yellow solid powder in yield 64.8 %，mp: 178-181 ℃.

^1^H NMR (400 MHz, DMSO*-d_6_*) *δ* 7.22 (*dd, J* = 5.3, 2.1 Hz, 1H), 7.20 (*d, J* = 5.2 Hz, 2H), 7.17 – 7.12 (*m,* 2H), 7.07 (*d, J* = 7.6 Hz, 1H), 6.94 (*td*, *J* = 7.7, 1.0 Hz, 1H), 6.86 – 6.81 (*m*, 1H), 6.65 (*d, J* = 7.5 Hz, 1H), 5.26 *(s*, 2H), 4.09 – 4.01 (*m*, 2H), 3.97 (*dd*, *J* = 6.6, 3.2 Hz, 1H), 2.96 (*s*, 2H), 2.52 – 2.48 (*m*, 2H), 1.99 (*dd*, *J* = 12.6, 2.8 Hz, 2H), 1.47 – 1.34 (*m*, 2H), 1.22 – 1.14 (*m*, 3H).

ESI-MS：([M+H]*^+^*) calcd for C_22_H_25_FN_4_O_2_：397.2，found：397.4.

**Synthesis of 1-(4-fluorobenzyl)-N-(piperidin-4-yl)-1H-benzo[d]imidazol-2-amine hydrobromide (3a)**

In a 50 mL round bottom flask, a mixture of intermediate **2a** (1.30 g, 3.28 mmol) and 37% hydrobromic acid was refluxed at 100 ° C for 15 hours. After the reaction was completed, the solution was concentrated in vacuo, and 20 mL of ethyl acetate was added for recrystallization to afford **3a** as a light gray solid in 73.1% yield, mp:261-263 ℃

^1^H NMR (400 MHz, DMSO*-d_6_*) *δ* 13.29 (*s*, 1H), 9.09 (*s*, 1H), 8.61 (*s*, 2H), 7.52 (*d*, *J* = 7.5 Hz, 1H), 7.43 (*d, J* = 8.0 Hz, 1H), 7.40 – 7.34 (*m*, 2H), 7.30 (*dd*, *J* = 16.6, 8.2 Hz, 2H), 7.23 (*dd, J* = 14.8, 6.0 Hz, 2H), 5.52 (*s*, 2H), 3.97 (*s*, 1H), 2.98 (*s*, 2H), 2.17 (*d, J* = 12.6 Hz, 2H), 1.88 (*d, J* = 10.8 Hz, 2H).

ESI-MS：([M+H]^+^) calcd for C_19_H_21_FN_4_：325.2，found：325.4.

**Synthesis of 2-(3-(4-((1-(4-fluorobenzyl)-1H-benzo[d]imidazol-2-yl)amino)piperidin-1-yl)propyl)isoindoline-1,3-dione(4a)**

In a 25 mL round bottom flask, a mixture of intermediate **3a** (0.10 g, 0.24 mmol) and N- (3-bromopropyl) phenylenediamine (0.15 g, 0.55 mmol) stirring well in 5 mL ethyl acetate 1 mL of DMF, anhydrous potassium carbonate (0.076 g, 0.55 mmol) was added, and refluxed at 80 ° C for 15 h. After the reaction was completed, water was added to the suspension, and the mixture was extracted with dichloromethane. The combined organic layers dried over Na_2_SO_4_, filtered, and concentrated in vacuo. Then, the crude product was purified by column chromatography to obtain **4a** as a transparent oil in 75.2% yield.

^1^H NMR (400 MHz, DMSO*-d_6_*) *δ* 7.87 (*dt*, *J* = 7.1, 3.7 Hz, 2H), 7.85 – 7.80 (*m*, 2H), 7.20 (*t, J* = 6.8 Hz, 2H), 7.18 – 7.13 (*m*, 2H), 7.05 (*d, J* = 7.7 Hz, 1H), 6.92 (*t, J* = 7.6 Hz, 1H), 6.82 (*t, J* = 7.6 Hz, 1H), 6.53 (*d, J* = 6.5 Hz, 1H), 5.77 (*s*, 1H), 5.26 (*s*, 2H), 3.64 (*t, J* = 6.9 Hz, 2H), 2.80 (*s*, 2H), 2.33 (*s*, 2H), 2.04 – 1.84 (*m*, 4H), 1.81 – 1.70 (*m*, 2H), 1.43 (*d, J* = 10.0 Hz, 2H).

ESI-MS：([M+H]^+^) calcd for C_30_H_30_FN_5_O_2_：510.6，found：512.4

**Synthesis of N-(1-(3-aminopropyl)piperidin-4-yl)-1-(4-fluorobenzyl)-1H-benzo[d]imidazol-2-amine(5a)**

In a 50 mL round-bottomed flask, the intermediate **4a**（0.42 g，0.82 mmol）and 5 mL of 80% hydrazine hydrate were stirred in 20 mL of absolute ethanol and refluxed at 80 ° C for 6 h. After the reaction was completed, the reaction solution was evaporated in vacuo to obtain a white solid and a yellow oil. The crude product was dissolved in dichloromethane, filtered, and the filtrate was evaporated in vacuo. The above operation was repeated until no white floc was produced, without further purification.

**Scheme S2.** Synthesis of E-4031-based pharmacophore

**Synthesis of N-(4-(1-acetylpiperidine-4-carbonyl)phenyl)methanesulfonamide(1b)**

In a 50 mL round-bottomed flask, 1-acetyl-4-piperidinecarboxylic acid (1.0 g, 5.84 mmol) was dissolved in 15 mL of 1,2-dichloroethane and preheated at 40 °C. Dichloro-sulfoxide (0.695 g, 5.84 mmol) was dissolved in 5 mL of 1,2-dichloroethane, added dropwise to a solution of 1-acetyl-4-piperidinecarboxylic acid, and refluxed at 65 °C for 1 h. After the reaction solution was cooled to room temperature, N-phenylmethanesulfonamide (1.0 g, 5.84 mmol) was added under ice bath conditions, anhydrous aluminum trichloride (2.8 g, 11.68 mmol) was added in portions, and stirred for 30 minutes on ice Under bath conditions, reflux at 80 ° C for 8 h. After the reaction, the reaction solution was poured into crushed ice. Then, it was extracted three times with dichloromethane when the crushed ice was melted. The organic layers were combined, dried over Na_2_SO_4_, filtered, and concentrated in vacuo. The crude product was purified by column chromatography to obtain **1b** as a white solid powder in 26.7% yield, mp: 209-211 ℃.

^1^H NMR (400 MHz, DMSO*-d_6_*) *δ* 10.34 (*s*, 1H), 8.00 (*d, J* = 8.6 Hz, 2H), 7.29 (*t, J* = 15.3 Hz, 2H), 4.39 (*d, J* = 13.0 Hz, 1H), 3.86 (*d, J* = 13.5 Hz, 1H), 3.64 (*ddd, J* = 11.3, 7.9, 3.7 Hz, 1H), 3.20 (*t, J* = 11.8 Hz, 1H), 2.73 (*t, J* = 11.5 Hz, 1H), 2.01 (*s*, 3H), 1.78 (*t, J* = 9.0 Hz, 2H), 1.58 – 1.45 (*m*, 1H), 1.41 – 1.29 (*m*, 1H).

ESI-MS：([M+H]^+^) calcd for C_15_H_20_N_2_O_4_S：325.1，found：325.4.

**Synthesis of N-(4-(piperidine-4-carbonyl)phenyl)methanesulfonamide hydrochloride(2b)**

In a 50 mL round bottom flask, the intermediate **2b** (0.50 g, 1.55 mmol) and 25 mL of 1M HCl were heated to 100 ° C and refluxed for 10 h. After the reaction was completed, the reaction solution was put into a refrigerator at 4 ° C. overnight, filtered, and the solid was washed with ethyl acetate to obtain **2b** as a light gray solid powder, in 75% yield, mp: > 270 ° C.

^1^H NMR (400 MHz, DMSO*-d_6_*) *δ* 10.43 (*s*, 1H), 9.16 (*s*, 1H), 8.84 (*s*, 1H), 8.00 (*d, J* = 8.7 Hz, 2H), 7.33 (*d, J* = 8.7 Hz, 2H), 3.77 – 3.65 (*m*, 1H), 3.30 (*d, J* = 12.5 Hz, 2H), 3.00 (*t, J* = 16.9 Hz, 2H), 1.92 (*d, J* = 12.3 Hz, 2H), 1.77 (*td, J* = 14.7, 3.7 Hz, 2H).

ESI-MS：([M+H]^+^) calcd for C_13_H_18_N_2_O_3_S：283.1，found：283.3.

**Scheme S3.** Synthesis of NBD-Cl derivatives

**Synthesis of N-(2-bromoethyl)-7-nitrobenzo[c][1,2,5]oxadiazol-4-amine(1d)**

In a 25 mL round bottom flask, a mixture of NBD-Cl (0.03 g, 0.15 mmol) and bromoethylamine (0.06 g, 0.30 mmol) was stirred in 10 mL of acetonitrile and anhydrous potassium carbonate (0.04 g, 0.30). mmol) was added. Then the mixture was warmed to 60 ℃ and refluxed for 5 h. After the reaction was completed, 30 mL of water was added to the reaction solution, and extracted with dichloromethane. The organic layers were combined, dried over Na_2_SO_4_, filtered, and purified by column chromatography to obtain **1d** as a red solid powder in 53.0 % yield.

^1^H NMR (400 MHz, DMSO*-d_6_*) *δ* 9.52 (s, 1H), 8.54 (*d, J* = 8.9 Hz, 1H), 6.59 (*t, J* = 26.4 Hz, 1H), 3.95 (*s*, 2H), 3.77 (*t, J* = 6.2 Hz, 2H).

**Synthesis of N-(3-bromopropyl)-7-nitrobenzo[c][1,2,5]oxadiazol-4-amine（2d）**

In a 50 mL round bottom flask, a mixture of NBD-Cl(0.09 g，0.45 mmol)and bromo-propylamine (0.09 g, 0.68 mmol) was stirred in 10 mL of acetonitrile and anhydrous potassium carbonate (0.04 g, 0.30). mmol) was added. Then the mixture was warmed to 60 ℃ and refluxed for 5 h. After the reaction was completed, 30 mL of water was added to the reaction solution, and extracted with dichloromethane. The organic layers were combined, dried over Na_2_SO_4_, filtered, and purified by column chromatography to obtain **1d** as a red solid powder in 80.2 % yield.

^1^H NMR (400 MHz, DMSO*-d_6_*) *δ* 9.54 (*d, J* = 5.4 Hz, 1H), 8.56 (*t, J* = 13.7 Hz, 1H), 6.45 (*d, J* = 9.0 Hz, 1H), 3.65 (*t, J* = 6.6 Hz, 2H), 3.61 (*s,* 2H), 2.30 – 2.18 (*m*, 2H).

**Synthesis of probe M1**

In a 50 mL round-bottomed flask, intermediate **7a** (0.07 g, 0.18 mmol) was dissolved in 20 mL of re-distilled 1,4-dioxane and stirred well. Then NBD-Cl (0.04 g, 0.18 mmol) and anhydrous potassium carbonate (0.05 g, 0.36 mmol) were added. The suspension was stirred at room temperature for 12 h. After the reaction was completed, filtered, evaporated to dryness in vacuum, add 10 mL of dichloromethane to dissolve the crude product, purified by column chromatography to obtain **M1** as an orange-red solid in 89.6% yield, mp:204-208 ℃.

^1^H NMR (400 MHz, DMSO*-d_6_*) *δ* 9.66 (*s*, 1H), 8.52 (*d, J* = 8.8 Hz, 1H), 7.21 (*dt, J* = 15.9, 7.9 Hz, 3H), 7.15 (*t, J* = 8.8 Hz, 2H), 7.07 (*d, J* = 7.7 Hz, 1H), 6.93 (*t, J* = 7.5 Hz, 1H), 6.84 (*t, J* = 7.5 Hz, 1H), 6.60 (*d, J* = 7.0 Hz, 1H), 6.44 (*d, J* = 9.0 Hz, 1H), 5.29 (*s*, 2H), 3.75 (*d, J* = 10.9 Hz, 1H), 3.55 (*s,* 2H), 2.90 (*d, J* = 9.9 Hz, 2H), 2.45 (*s*, 2H), 2.17 – 1.94 (*m*, 4H), 1.93 – 1.80 (*m*, 2H), 1.62 (*d, J* = 10.0 Hz, 2H).

^13^C NMR (100MHz, DMSO*-d_6_*) *δ* 163.03, 160.62, 154.41, 145.78, 144.97, 143.29, 138.38, 134.73, 133.90, 129.48, 120.95, 118.83, 115.91, 115.70, 115.48, 108.28, 99.57, 55.75, 52.77, 50.49, 44.11, 42.63, 32.26, 25.37.

HRMS (ESI) calcd. for C_28_H_29_FN_8_O_3_[M+H]^+^ 545.2425, found 545. 2423.

HPLC t_R_ =12.9 min, 98.1%, silica column, mobile phase: methanol-water (80:20, v/v), λ = 468 nm.

**Synthesis of probe M2**

A mixture of intermediate **2** (0.03 g, 0.10 mmol) and intermediate **1d** (0.02 g, 0.07 mmol) was stirred in 10 mL of DMF, and sodium carbonate (0.015 g, 0.14 mmol) was added, and the mixture was refluxed at 70 ° C for 24 h. After the reaction was completed, 50 mL of water was added to the reaction solution, and dichloromethane was added for extraction. The organic layers were combined, dried over NaSO_4_, filtered, and concentrated in vacuo. The crude product was purified by column chromatography to obtain **M2** as an orange solid power in 40.9% yield, mp:202-204 ℃.

^1^H NMR (400 MHz, DMSO*-d_6_*) *δ* 10.23 (*s*, 2H), 8.07 – 7.99 (*m*, 2H), 7.96 (*d, J* = 8.7 Hz, 1H), 7.76 – 7.64 (*m*, 1H), 7.30 (*dd, J* = 8.5, 5.3 Hz, 2H), 3.70 (*dd, J* = 22.3, 10.5 Hz, 1H), 3.66 – 3.56 (*m*, 1H), 3.25 – 3.16 (*m*, 1H), 3.12 (*d, J* = 3.3 Hz, 3H), 2.98 (*t, J* = 13.2 Hz, 1H), 2.81 (*td, J* = 12.7, 2.9 Hz, 1H), 2.68 (*t, J* = 6.4 Hz, 1H), 2.21 (*t, J* = 10.9 Hz, 1H), 2.05 – 1.92 (*m*, 1H), 1.80 (*dd, J* = 30.7, 16.5 Hz, 2H), 1.64 (*dd, J* = 14.6, 6.8 Hz, 1H), 1.44 – 1.33 (*m*, 2H).

^13^C NMR (100 MHz, DMSO*-d_6_*) *δ* 201.50, 167.44, 143.45, 132.08, 130.72 (s), 130.44 (s), 129.14, 118.11, 65.50, 53.08, 42.72, 31.76, 30.48, 29.50, 28.93, 27.02, 22.56, 19.12, 14.02.

HRMS (ESI) calcd. for C_21_H_24_N_6_O_6_S[M+H]^+^ 489.1556, found 489.1540.

HPLC t_R_ =5.4 min, 97.8%, silica column, mobile phase: methanol-water (65:35, v/v), λ = 471 nm.

**Synthesis of probe M3**

A mixture of intermediate **2** (0.05 g, 0.16 mmol) and intermediate 2d (0.05 g, 0.16 mmol) was stirred in 10 mL of DMF, and sodium carbonate (0.008 g, 0.08mmol) was added, and the mixture was refluxed at 70 ° C for 24 h. After the reaction was completed, 50 mL of water was added to the reaction solution, and dichloromethane was added for extraction. The organic layers were combined, dried over NaSO_4_, filtered, and concentrated in vacuo. The crude product was purified by column chromatography to obtain **M3** as an orange solid power in 24.9% yield, mp:204-206 ℃.

^1^H NMR (400 MHz, DMSO*-d_6_*) *δ* 10.14 (*s*, 2H), 8.52 (*d, J* = 8.7 Hz, 1H), 7.98 (*dd, J* = 14.9, 6.6 Hz, 2H), 7.30 (*d, J* = 8.6 Hz, 2H), 6.43 (*d, J* = 9.0 Hz, 1H), 3.52 (*d, J* = 25.7 Hz, 2H), 3.11 (s, 3H), 2.96 (*d, J* = 10.9 Hz, 2H), 2.47 (*t, J* = 6.5 Hz, 2H), 2.10 (*t, J* = 11.0 Hz, 2H), 2.05 – 1.92 (*m*, 1H), 1.88 (*dd, J* = 13.0, 6.5 Hz, 2H), 1.76 (*d, J* = 12.1 Hz, 2H), 1.72 – 1.57 (*m*, 2H).

^13^C NMR (101 MHz, DMSO*-d_6_*) *δ* 201.43, 145.70, 144.77, 143.73, 138.36, 130.62, 130.41, 120.98, 118.16, 99.56, 56.16, 55.37, 53.10, 42.91, 28.92, 24.95.

HRMS (ESI) calcd for C_22_H_26_N_6_O_6_S[M+H]^+^ 503.1713, found 503.1705.

HPLC t_R_ =4.9 min, 98.9%, silica column, mobile phase: methanol-water (70:30, v/v), λ = 474 nm.

1. **Fluorescent Properties of Probes Assay**

Probes **M1-M3** were prepared into 10 mM concentrated stocks with DMSO, and diluted into 5 μM、10 μM、20 μM、40 μM and 80 μM solution with PBS (pH = 7.4). And dilute the stock solution to 10 μM with acetonitrile, anhydrous methanol, water, and dichloromethane. The fluorescent properties of probes were obtained on PerkinElmer EnSight microplate reader.

The quantum yield of the probes **M1-M3** in PBS solution (pH = 7.4) is obtained by comparison with fluorescein in 0.1 M NaOH (ΦST = 0.92). The calculation formula is as follows: Φ_X_ = Φ_ST_ (A_ST_ /A_X_) (F_X_ /F_ST_) (ƞ_X_ /ƞ_ST_)^2^. Where the subscripts ST and X denote standard and test products, Φ is the quantum yield, F is the integrated area under the fluorescence emission spectrum, A is the absorbance, and ƞ is Refractive index of the solvent. Determination of Quantum Yield by Steady State / Transient Fluorescence Spectrometer (FLS920)

**hERG Potassium Channel Binding Assay**

**Fig. S1** Competitive binding curve of probe M1-M3 and astemizole to hERG potassium channel

To use small-molecule fluorescent probes for practical cell imaging, it is necessary to have a high affinity for hERG potassium ion channels. Shanghai Huiyuan Biotechnology Co., Ltd.). In this experiment, astemizole, which has a high affinity for hERG potassium channels, was selected as the positive control drug. Based on the radioligand 3H-dofetilide (7.5 nM), small molecule probes (**M1-M3**) were used to determine Inhibitory activity. The small molecule probe was prepared into a 10 mM concentrated stock solution with DMSO, and then bound with Assay Buffer (10 mM HEPES, pH 7.4, 130 mM NaCl, 60 mM KCl, 0.8 mM MgCl2, 10 mM glucose, 1 mM EGTA, 0.1 % BSA) was diluted to 10 concentration gradients, and two replicates were set for each concentration. Then, 80 μL hERG-HEK293 cell membrane (10 μg / well), 10 μL ^3^H-dofetilide (final concentration 7.5 nM), and 10 μL small molecule probe solution were added to UniFilter-96 (GF / B) incubate in a microtiter plate at 37 °C for 1.5 h. Stopping the reaction by quickly filtering the UniFilter-96 (GF / B) microplate in the above (2) reaction with a cell collector. The microtiter plate was washed three times with Tris-HCl buffer (pH 7.4), and dried at 37 °C for 30 min. 50 μL of scintillation solution was added to each well. Finally, the MicroBeta Trilux microplate scintillation luminometer was used to determine the radiological count (1 min) of bound 3H-dofetilide in each well.

1. **Cytotoxicity**

The cytotoxicity effects of probes were determined by SRB assays, using hERG transfected HEK293 cells (or hERG-HEK293, purchased from Shanghai Genechem Co., Ltd). An amount of 8000 cells per well were seeded in 96-well plates in 100 μL culture medium and cultured in 5% CO_2_ atmosphere at 37 °C for 24 h. Then, the cells were treated with 100 μL of different concentrations solutions of each probe (**M1**, **M2**, **M3**) for 24 h, respectively. 50 μL 50% TCA (pre-cooled at 4 °C) was added to each well to fix the cells and left at 4 °C for 1 h. Then, each well was washed with distilled water to remove TCA and dried at room temperature. 100 μL of 4 g / L SRB solution was added to each well and shaken for 20 min on a constant temperature shaker (37 °C) to stain, washed with 1% acetic acid to remove SRB and dried at room temperature. Subsequently, 100 μL of Tris (pH = 10.5) solution was added to each well, shaken for 15 min on a constant temperature shaker (37 °C), and the absorbance at 540 nm was measured with a microplate reader (POLARstar Omega). Calculate the inhibition rate according to the following formula: Inhibition rate% = (1- (Absorbance of the dosing group-absorbance of the blank control group) / (absorbance of the negative control group-absorbance of the blank control group)) × 100%, and IC_50_ was calculated by GraphPad Prism 6.

1. **Microscopic imaging of hERG-HEK293 cells**(Liu et al. 2015)

hERG-HEK293 cells (purchased from Shanghai Genechem Co., Ltd) were grown in DMEM medium supplemented with 10% (v / v) fetal bovine serum in 5% CO2 and 95% air at 37 ° C. The cells were plated on a confocal dish and allowed to stabilize for 24 hours. After aspirating the medium, the cells were carefully washed with PBS buffer solution, and 1 mL of DMEM medium without fetal bovine serum was added, followed by incubation at 37 ° C for 10 minutes. Or in the presence of probes **M1-M3** (prepared in DMEM without fetal bovine serum), or co-incubate with **M1-M3** and astemizole for 10 minutes at 37 ° C. Fluorescence imaging was performed using a Zeiss Axio Observer A1 fluorescence microscope.


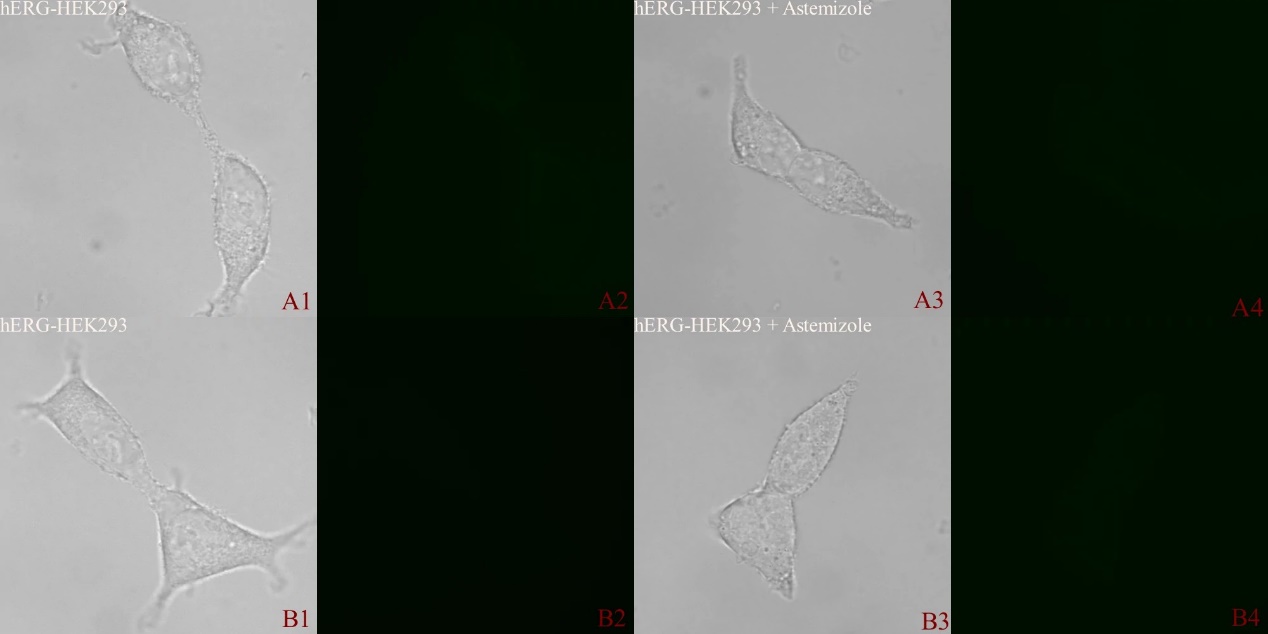


**Fig. S2** Autofluorescence imaging of hERG-HEK293 cells in the presence or absence of astemizole. A1, A3, B1, B3: bright field; A2, A4, B2, B4: GFP channel. A3, A4: Astemizole (10 μM); B3, B4: Astemizole (50 μM); A1, A2, B1, B2 blank group (DMEM medium without fetal bovine serum). Scale bar = 20 μm.

1. **1H-NMR, 1^3^C-NMR,** **ESI-HRMS**

**Fig. S3** ^1^H-NMR spectrum, ^13^C-NMR spectrum, and ESI-HRMS spectrum of compound M1

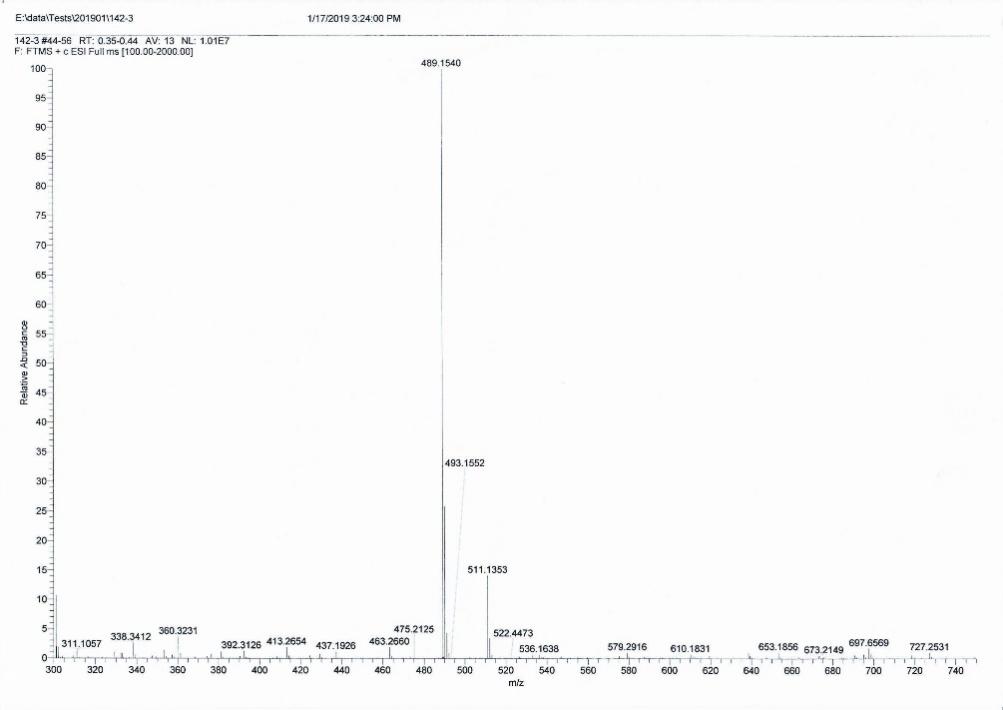


**Fig. S4** ^1^H-NMR spectrum, ^13^C-NMR spectrum, and ESI-HRMS spectrum of compound M2

**Fig. S5** ^1^H-NMR spectrum, ^13^C-NMR spectrum, and ESI-HRMS spectrum of compound M3

1. **References**

Liu Z, Wang B, Ma Z, Zhou Y, Du L, Li M. 2015. Fluorogenic Probe for the Human Ether-a-Go-Go-Related Gene Potassium Channel Imaging. Anal Chem (Washington, DC, U S). //;87:2550-2554.

Wang B, Liu Z, Ma Z, Li M, Du L. Astemizole Derivatives as Fluorescent Probes for hERG Potassium Channel Imaging. Acs Medicinal Chemistry Letters.acsmedchemlett.5b00360.

Zhang X, Liu T, Li Q, Li M, Du L. Aggregation-Induced Emission: Lighting Up hERG Potassium Channel. Front Chem.7:54
